# Impact of telephone triage on emergency after hours GP Medicare usage: a time-series analysis

**DOI:** 10.1186/1743-8462-4-21

**Published:** 2007-10-10

**Authors:** David Dunt, Robert Wilson, Susan E Day, Margaret Kelaher, Lyle Gurrin

**Affiliations:** 1 Centre for Health Policy, Programs and Economics, School of Population Health, University of Melbourne, 3010 Vic Australia; 2 Robert H Wilson & Associates Pty Ltd, Health Economics Consultants, 4 Avoca St (off Toorak Rd) South Yarra 3141 Vic Australia; 3 Centre for Molecular, Environmental, Genetic and Analytic Epidemiology, School of Population Health, University of Melbourne, Melbourne, 3010 Vic Australia

## Abstract

**Background:**

The Australian government sponsored trials aimed at addressing problems in after hours primary medical care service use in five different parts of the country with different after hours care problems. The study's objective was to determine in four of the five trials where telephone triage was the sole innovation, if there was a reduction in emergency GP after hours service utilization (GP first call-out) as measured in Medicare Benefits Schedule claim data. Monthly MBS claim data in both the pre-trial and trial periods was monitored over a 3-year period in each trial area as well as in a national sample outside the trial areas (*National comparator*). Poisson regression analysis was used in analysis.

**Results:**

There was significant reduction in first call out MBS claims in three of the four study areas where stand-alone call centre services existed. These were the *Statewide Call Centre *in both its *Metropolitan *and *Non-metropolitan *areas in which it operated – Relative Risk (RR) = 0.87 (95% Confidence interval: 0.86 – 0.88) and 0.60 (95% CI: 0.54 – 0.68) respectively. There was also a reduction in the *Regional Call Centre *in the non-Metropolitan area in which it operated (RR = 0.46 (95% CI: 0.35 – 0.61) though a small increase in its *Metropolitan area *(RR = 1.11 (95% CI: 1.06 – 1.17). For the two telephone triage services embedded in existing organisations, there was also a significant reduction for the *Deputising Service *– RR = 0.62 (95% CI: 0.61 – 0.64) but no change in the *Local Triage centre *area.

**Conclusion:**

The four telephone triage services were associated with reduced GP MBS claims for first callout after hours care in most study areas. It is possible that other factors could be responsible for some of this reduction, for example, MBS submitted claims for after hours GP services being reclassified from 'after hours' to 'in hours'. The goals of stand-alone call centres which are aimed principally at meeting population needs rather than managing demand may be being met only in part.

## Background

This paper reports further on the national evaluation of the After Hours Primary Medical Care Trials (AHPMCTs) which were a recent initiative of the Australian Government. The goals of these trials were to improve the quality of service delivery as well as consumer acceptability, consumer access (including affordability) and equity, appropriateness of service mix, provider satisfaction with regard to their impact on service mix as well as service use more generally [1].

The common feature of these trials was the use of telephone triage. Telephone triage and advice services have an important place in the development of after hours care in other countries. Most frequently these services are embedded in other after hours services such as in GP cooperatives in the UK, HMOs in the US and the county-based service arrangements in Denmark introduced in the 1990s [2]. A small number of stand-alone services have also been established. NHS Direct is a 24 hour, confidential telephone, online and interactive digital TV health advice and information service provided by the National Health Service in England and Wales (similar service in Scotland). NHS Direct was rolled out across England and Wales between 1998 and 2000. The telephone service aims to triage symptomatic callers to provide guidance on which healthcare provider the caller should access. Nurses using proprietary health call centre software also give advice on how to manage an episode of illness at home. Health Information Advisors can provide information on a wide range of medical conditions, treatments, medicines and NHS services. In some areas of England and Wales, NHS Direct is commissioned by local Primary Care Trusts (PCTs) to provide the gateway for out-of-hours access to GP's surgeries and clinics. [3].

A structured review on the impact of after hours GP services on clinical outcomes, medical workloads as well as patient and GP satisfaction concluded that this growth in the use of telephone triage and advice services usually, but not always reduced immediate medical workload through the substitution of telephone consultations for face-to-face consultations [4]. For example, a before and after study following the introduction of NHS Direct as a stand-alone service in the UK found a small, but significant reduction in use of GP co-operatives but no change in use of Emergency Department (ED) and ambulance services in the study area [5]. Considering embedded services, a randomised controlled trial from the UK compared a nurse telephone consultation service integrated within a GP co-operative with the usual practice of that co-operative [6]. There was a 69% reduction in telephone advice from a GP, a 38% reduction in patient attendance at primary care centres and a 23% reduction in home visits.

Previous research on the APHMCTs using population-based telephone survey data of after hours service utilisation found that the introduction of telephone triage services, whether embedded in other services or stand-alone, was not clearly associated with reduction in after hours use or shift towards the use of GP after hours clinics [7]. It is important therefore to extend analysis to include not only self-reported data, but also administrative data routinely recorded on service utilisation. This study presents Medicare Benefits Schedule (MBS) data from Medicare Australia (formerly the Health Insurance Commission [HIC]) bearing in mind that this data too had limitations. At the time of the study, MBS data recorded only GP emergency (first doctor call-out whether to consulting rooms or patient's home) after hours service use and not all other routine after hours GP use. Thus the data forms one small but important part of all after hours service use.

The aim of this study then is to determine if there is a reduction in emergency (GP first callout) after hours service use recorded in MBS data following the introduction of different forms of telephone triage in the AHPMCTs, where these were the sole service innovation.

### The After Hours Primary Medical Care Trials

Telephone triage services used by the four study AHPMCTs varied both in the form of the telephone triage service offered and its service function within the individual AHPMCT as follows [1].

#### Stand-alone services

These were call centres where nurses, using proprietary health call centre software, aimed at providing more accessible advice and promoting more appropriate after hours primary medical care (PMC) service use. They were:

• the stand-alone *Statewide Call Centre *(studied in both *Metropolitan *and *Non-metropolitan *areas); and

• the stand-alone *Regional Call Centre *(studied in both inner metropolitan (*Metropolitan*) and rural satellite areas (*Non-metropolitan*);

#### Embedded services

These triage and advice systems did not use proprietary health call centre software. They aimed more at managing demand to support the GP workforce in terms of recruitment and retention. They were:

• a GP-based telephone triage and advice service (without guidelines or software) in a well-established, pre-existing *Deputising Service;*

• the *Local Triage *and advice service using hospital nurses with locally developed, paper-based protocols to support after hours services in GP own-practice arrangements in a small rural community.

The AHPMCT services together with the regional context of the trials and their organisational settings are summarised in Table 1. It should be noted that while the *Statewide Call Centre *received funding as part of the AHPMCTs this was mainly for logistic and information support. The service opened a short time before the other AHPMCTs with separate Australian and State government funding. While it provides 24-hour advice on a variety of health-related topics, its involvement in after hours primary care is the focus of this paper. The AHPMCTs commenced operation during 1999–2000. Variation in the date of commencement affected whether or not one or two years of pre-trial data was available. It also meant, for a few trials, that data covered less than 12 months of operation, introducing unavoidable seasonal bias.

**Table 1:** Local context and services of individual trials.

**Trial**	Catchment Areas *^†^	**Frequency of telephone calls to service (calls/month/10,000 head of population) [1]**	**Notes**
1. Statewide Call Centre	Capital city, small rural centre and Other rural area 1.42M (Observation period for trial Feb99–Jan00 and Feb00–Dec00). Rest of state – small rural centre and other rural area studied 42,500 (Observation period for trial July00–Dec00)	50 (Metropolitan) Data not available (Non-metropolitan)	1 Operator with prior international industry experience under contract to the State Government) – phase-in period when calls to a public hospitals were transferred to the call centre 2 Free to caller 3 Coincidentally, a number of capital city after hours walk-in GP clinics opened around the time the *Statewide Call Centre *was established.
2. Regional Call Centre	Capital city (inner region) 537,000 Non-metropolitan satellite: Small rural centre 21,000 (Observation period for trial May00–Dec 00)	18 (Metropolitan) 6 (Non-metropolitan)	1 Startup operator – phase-in period when calls to two public hospitals were transferred to the call centre 2 Free to caller 3 A GP after hours walk-in clinic operating with no fee to patients located adjacent to the ED of a regional hospital in the central metropolitan area operating for part of the study period but closed due to lack of demand. 4 Prior restricted access to after hours GP care in non-metropolitan satellite
3. Deputising Service	Capital city and Other rural area 229,000 (Observation period for trial – October 1999 to September 2000)	31 (approx)	1 GP-based telephone triage and advice service 2 Pre-existing and continuing *Deputising Service *offering an after hours walk-in clinic and home visiting service. 3 Service offered to patients of previously enrolled GP practices (85% of all practices in capital city) and new enrolled GP practices beyond the metropolitan area (52% practice take-up). 4 Commonwealth government funding of the program derived from 'cashing out' historical MBS reimbursements to the service – home visits by *Deputising Service *staff to patients in capital city (only) during the trial not billed to patient.
4. Local Triage and advice service	Other rural area (ie small country town and surrounding community) 21,000 (Observation period for trial – October 1999 to September 2000)	24 (approx)	1 Hospital nurses using locally developed paper-based protocols 2 Located in a local hospital in a rural community. 3 Aim to support GP own-practice after hours arrangement to relieve GP after hours burden.

* Based on Rural, Remote and Metropolitan Areas classification (RRMA) [11]

† Source: Australian Bureau of Statistics population projections for 1999 supplied to the Department of Health and Ageing

## Results

### National comparator – see Table 2

**Table 2:** Utilisation rates of MBS services (per 10,000 population): National comparator.

**TIME PERIOD (POPULATION)**	**IN-HOURS**	**AFTER HOURS**	**VR GPS**				**NON VR GPS**			
			
			**Not "UNSOCIABLE" HOURS**		**"UNSOCIABLE" HOURS**		**NOT "UNSOCIABLE" HOURS**		**"UNSOCIABLE" HOURS**	
			
			**Not CON ROOMS**	**CON ROOMS**	**NOT CON ROOMS**	**CON ROOMS**	**NOT CON ROOMS**	**CON ROOMS**	**Not CON ROOMS**	**CON ROOMS**
			
			**1**	**2**	**601**	**602**	**97**	**98**	**697**	**698**
Oct97/Sep98 (16,344,730)	4025.3	30.0	11.0	4.2	2.0	0.7	7.8	2.2	1.9	0.2
Oct98/Sep99 (16,517,748)	3975.5	29.2	11.4	4.1	2.4	0.9	6.4	1.5	2.3	0.4
Oct99/Sep00 (16,689,060)	3871.7	28.0	10.7	3.9	2.0	1.0	6.3	1.3	2.4	0.4

**Notes: **For the non-trial areas the period October 1997 to September 2000 was analysed as this represented the most common period over which the trials were conducted. Sociable hours (8–11 pm)and unsociable hours (11 pm–8 am)

After hours utilisation rates were very small in number compared to in-hours utilisation rates. VR GPs provided most after hours services (59.3% in 97/98, 62.9% in 99/00). Most after hours services were provided in sociable hours (84.0% in 97/98, 79.3% in 99/00) and outside consulting rooms (75.7% in 97/98, 76.4% in 99/00). Services provided by non-VR GPs, compared to VR GPs were most common in unsociable hours.

There was a 6.7% reduction in after hours utilisation rates from 30.0 to 28.0 per 10,000 population per month across the study period outside of the trial areas. There was also a 3.8% decline in the in-hours utilisation rate during this time. The decline in number of after hours services occurred more in the consulting room (9.6%) than outside (5.7%). This decline also occurred in sociable hours (8–11 pm) (11.9%) but not in unsociable hours (11 pm–8 am) which increased substantially (20.8%).

### Stand-alone services – see Tables 3, 4, 5, 6

**Table 3:** Utilisation rates of MBS services (per 10,000 population): AHPMCT stand-alone services – Statewide Call Centre (Metropolitan).

Feb98/Jan99 (1,398,781)	3920.6	19.1	4.8	0.7	1.2	0.0	8.5	0.6	3.3	0.0
**Trial period begins**										

Feb99/Jan00 (1,424,036)	3881.6	16.3	4.9	0.7	1.3	0.0	6.4	0.5	2.6	0.0
Feb00/Dec00 (1,449,729)	3790.0	15.7	3.9	0.5	0.8	0.0	7.5	0.2	2.6	0.0

**Table 4:** Utilisation rates of MBS services (per 10,000 population): AHPMCT stand-alone services – Statewide Call Centre (Non-metropolitan).

Jul98/Jun99 (118,162)	2892.9	15.1	10.2	2.1	1.4	0.2	1.0	0.3	0.0	0.0
Jul99/Jun00 (120370)	2870.9	9.6	4.7	3.4	0.7	0.1	0.7	0.2	0.0	0.0
**Trial period begins**										

Jul00/Dec00 (122,211)	2810.1	8.6	4.0	2.5	0.5	0.1	1.1	0.4	0.0	0.0

**Table 5:** Utilisation rates of MBS services (per 10,000 population): AHPMCT stand-alone services – Regional Call Centre (Metropolitan):

May 98/Apr99 (532,478)	3548.0	6.6	2.8	0.8	0.3	0.2	1.6	0.8	0.0	0.1
May99/Apr00 (536,886)	3468.4	6.6	3.2	0.9	0.4	0.1	1.5	0.5	0.0	0.0
**Trial period begins**										

May99/Dec00 (539,030)	3473.4	6.6	3.4	1.0	0.2	0.2	1.0	0.7	0.0	0.0

**Table 6:** Utilisation rates of MBS services (per 10,000 population): AHPMCT stand-alone services – Regional Call Centre (Non-metropolitan):

May98/Apr99 (21,261)	3927.8	12.7	3.3	1.4	0.0	0.0	8.0	0.0	0.0	0.0
May 99/Apr00	3468.1	8.1	5.2	0.5	0.0	0.0	2.4	0.0	0.0	0.0
**Trial period begins**										

May 00/Dec00	3058.0	5.3	2.9	0.5	0.0	0.0	1.9	0.0	0.0	0.0

#### Statewide Call Centre – Metropolitan

The total monthly after hours utilisation rate in the area was low – approximately 63.7% of rates elsewhere in Australia judged by the *National Comparator*.

There was a 17.8% reduction in total monthly after hours utilisation from 19.1 to 15.7 per 10,000 population per month following the introduction of the AHPMCT. The decline in number of in-hours service by comparison was quite small (2.9%). Reduction in both after hours consulting room and non-consulting room services was substantial (7.7% and 14.6% respectively) in the first 12 months. There was a further large reduction in consulting room services (41.7%) but not non-consulting room (2.6%) in the following 11 months. A higher proportion of after hours services were outside of the consulting room (93.2 – 95.5%) compared with 75.7 – 76.4% in the National comparator area.

Following the introduction of the AHPMCT, there was a significant reduction in the total monthly after hours utilisation rate (RR = 0.87 (95% CI: 0.86 – 0.88)) when compared with the *National comparator*.

#### Statewide Call Centre – Non-metropolitan

The total monthly after hours utilisation rate in the area was low, 50.3% of rates elsewhere in Australia judged by the *National Comparator*. After hours services were predominantly supplied by VR GPs (in excess of 80%). Very few (less than 10%) of total after hours services were supplied in unsociable hours and all of these were supplied by VR GPs.

There was a 36.4% reduction in total monthly after hours utilisation rate from 15.1 to 9.6 per 10,000 population per month after the introduction of the *Statewide Call Centre*. In-hours items were relatively stable across the 3-years period by comparison. Non-consulting room services declined from 82.9% to 65.1% as a proportion of total after hours services over the 3-year period (compared to 75.7 – 76.4% nationally outside the trial areas).

Following the introduction of the AHPMCT, there was a significant reduction in the total monthly after hours utilisation rate (RR = 0.60, 95% CI: 0.54 – 0.68) when compared with the *National comparator*.

#### Regional Call Centre – Metropolitan

The total monthly after hours utilisation rate in the area was low – approximately 23% of rates elsewhere in Australia judged by the *National Comparator*. The total monthly after hours utilisation rate remained steady at 6.6 per 10,000 population per month following the introduction of the *Regional Call Centre*. Services in and out of the consulting room also remained steady.

Following the introduction of the AHPMCT, there was a small increase in the total monthly after hours utilisation rate *(*RR = 1.11, 95% CI: 1.06 – 1.17) when compared with the *National comparator*.

#### Regional Call Centre – Non-metropolitan

The total monthly after hours utilisation rate in the area was low – approximately 42.3% of rates elsewhere in Australia judged by the *National Comparator*.

The total monthly after hours utilisation rate reduced by 58.3% from 12.7 to 5.3 per 10,000 population per month after the introduction of the AHPMCT. Following the introduction of the AHPMCT there was a significant reduction in the total monthly after hours utilisation rate (RR = 0.46, 95% CI: 0.35 – 0.61) when compared with the *National comparator*.

### Embedded services – see Tables 7, 8

**Table 7:** Utilisation rates of MBS services (per 10,000 population) – AHPMCT embedded services – Deputising Service.

Oct97/Sep98 (229,593)	3888.5	39.9	9.6	12.7	2.2	0.5	7.1	5.4	2.3	0.1
Oct98/Sep99 (229,223)	3799.4	35.4	8.6	12.3	1.8	0.5	4.9	4.4	2.7	0.1
Trial period begins*										

Oct99/Sep00 (229,019)	3721.4	22.3	5.8	10.9	0.6	0.3	1.4	3.2	0.1	0.2

* excludes unbilled services provided by the *Deputising Service*. If these are included there were 24.3 per 10,000 after hours services provided in Oct99/Sep00. Data including for unbilled services not available for other than total after hours services.

**Table 8:** Utilisation rates of MBS services (per 10,000 population) – AHPMCT embedded services – Local Triage centre.

Oct97/Sep98 (21,395)	2467.4	49.5	35.5	0.5	4.7	0.0	7.5	0.5	0.9	0.0
Oct98/Sep99 (21,309)	2488.6	43.2	32.8	0.5	5.6	0.0	4.2	0.0	0.5	0.0
**Trial period begins**										

Oct99/Sep00 (21,259)	2510.5	44.7	31.5	0.5	5.6	0.0	5.6	0.0	0.9	0.0

#### Deputising Service

The total monthly after hours utilisation rate in the area was approximately 33% higher than rates elsewhere in Australia judged by the *National Comparator*.

There was a 39.1% reduction in total monthly after hours utilisation rate from 39.9 to 24.3 per 10,000 population per month after the introduction of the AHPMCT. The decline in number of in-hours service by comparison was quite small in comparison. Following the introduction of the AHPMCT there was a significant reduction in the total monthly after hours utilisation rate (RR = 0.62, 0.61 – 0.64) when compared with the *National comparator*. This excluded the unbilled services provided by the *Deputising Service *(2.0 per 10,000 population per month), the effects of which should be very small.

#### Local Triage centre

The total monthly after hours utilisation rate in the *Local Triage centre *area was 65.0% higher than rates elsewhere in Australia judged by the *National Comparator*.

There was a 9.7% reduction in total monthly after hours utilisation rate from 49.5 to 44.7 per 10,000 population per month after the introduction of the AHPMCT. (Rates and change in rates should be interpreted cautiously as numbers were small and confidence intervals wide.) Nearly all after hours services (98–99%) were delivered outside the consulting room and this did not change during the trial (compared to 76–77% in the *National Comparator area*. Following the introduction of the AHPMCT the total monthly after hours utilisation rate did not change (RR = 1.01, 0.94 – 1.08) and when compared with the *National comparator*.

## Discussion

Considering first stand-alone services, there was a significant reduction in after hours utilisation in three of the four study areas – the *Statewide Call Centre *in both *Metropolitan *and *Non-metropolitan *areas, (larger in the *Non-metropolitan *area) as well as the *Non-metropolitan *areas in the *Regional Call Centre*. (There was a small increase in the *Metropolitan area*). Considering embedded services, there was a significant reductionin after hours utilisation following the introduction of the *Deputising Service *but no change in the *Local Triage centre *area. Thus, after hours utilisation rates decreased in four of the six AHPMCT areas (three substantially) – there was no change in one area and a small increase in the other.

The variation in after hours service utilisation rate in the six areas at baseline from 49.5 per 10,000 monthly in the *Local Triage centre *to 6.6 per 10,000 per month in the *Metropolitan *area of the *Regional Call Centre *is noteworthy (after hours service utilisation rate was 30.0 per 10,000 monthly in the rest of Australia at that time).

Some limitations of the study should be acknowledged. As noted, the impact of the trials was monitored using monthly data before and after the introduction of the trials taking into account secular trend at the national level only. This is a relatively weak study design However it was not possible nor intended to compare the success of individual trials which had different health service characteristics, and resident population characteristics and consequently population health needs, as well as somewhat different service aims and arrangements. In general, local data collection at EDs and ambulance services was inconsistently recorded and it was not possible to estimate robustly the effects of the AHPMCTs on these services. Immediate after hours GP service was only studied – not subsequent in-hours GP or specialist use. Finally, as noted, only emergency GP after hours usage was identified in the MBS.

Some possible explanations for these changes in after hours service utilisation, other than impact of the AHPMCTs need to be considered. In the *Statewide Call Centre – Metropolitan area*, a number of new after hours clinics, unrelated to the AHPMCT, set up operation at the same time as the AHPMCT. They could be expected therefore to have effects on after hours utilisation rates that were independent of the AHPMCT. These clinics typically provided non-emergency services not reimbursed in the MBS as an after hours care item, and their effects would not be recorded as an (emergency) after hours items. However some effect on emergency after hours use and some confusion with AHPMCT effects are possible. Since the completion of the AHPMCTs, a new MBS item has been introduced to reimburse routine after hours service use. Its existence during the trial would have provided greater clarity concerning this issue as the phenomenon of shift in after hours GP care from emergency (ie first callout) after hours care and identified as such to non-emergency (ie after first callout) and not identified as after hours would be common.

In the *Deputising Service *a 'cashing-out' arrangement was put in place – after hours GPs were paid on a salary basis, patients paid no fee for a home visit. This shift in payment arrangement from fee-for-service to salary rather than telephone triage could have been responsible for reducing utilisation.

In all AHPMCTs, the decrease in after hours utilisation rates after the introduction of the AHPMCT could be due to other factors unrelated to the AHPMCT, such as an acceleration of the longer-term decline in after hours utilisation rates in the trial areas. As the AHPMCTs were established in response to local needs and difficulties in relation to after hours services, it is possible that these local needs and difficulties continued after the establishment of the AHPMCT. For example in the *Non-metropolitan area *of the *Regional Call Centre *there had been a crisis in the provision of after hours services with almost no unsociable hours care being provided by GPs.

Some patients may have crossed service boundaries to receive their after hours care and this was not recorded in our data collection system. We only have estimates of the magnitude of the opposite phenomenon where patients beyond the boundaries received their after hours care in AHPMCT areas. This was measured at around 5% of all local GP usage in AHPMCT areas (and included in our analysis). While this phenomenon could introduce some 'noise', it should not affect the validity of results.

In assessing to what extent the AHPMCTs were responsible for the changes in GP after hours utilisation, it is worthwhile to consider to what extent there was take-up of AHPMCT services. The telephone triage call rate in the four AHPMCTs varied between 50 and 6 after hours calls/month/10,000 population in the Metropolitan area of the *Statewide Call Centre *and the Non-metropolitan area of the *Regional Call Centre *respectively (possibly related to the level of marketing and promotion for the service). The uptake of telephone triage, with the exception of the *Regional Call Centre *(Non-metropolitan area), *prima facie *should have been sufficient to produce an effect on utilisation levels and service mix.

It is also worthwhile to consider to what extent these MBS after hours utilisation results were consistent with those reported in our earlier population survey study population [5]. Stand-alone services did not align well. There was a significant reduction in after hours GP service usage in the MBS dataset but a significant increase in the population dataset in the *Statewide Call Centre – Metropolitan *area. This might be explained as an effect of the opening of the after hours GP clinics unrelated to the AHPMCT in Perth with this extra activity generating additional MBS in-hours rather than after hours item numbers as these would not have been emergency in nature and not recorded as an after hours MBS item. There was a significant reduction in after hours GP service usage in the MBS dataset in the *Statewide Call Centre – Non-metropolitan area *but no change in the population dataset. There was a small significant increase in after hours GP service usage in the MBS dataset in the *Regional Call Centre – Metropolitan *area but no change in the population dataset. There was a significant reduction in after hours GP service usage in the MBS dataset but a significant increase in the population dataset in the *Regional Call Centre – Non-metropolitan area*.

MBS results for embedded services aligned better with population results. There was a reduction for MBS service use and less GP clinic use (both per person contact and frequency) in the population survey for the *Deputising Service*. There was no change in use reported in both MBS and population survey dataset for the *Local Triage centre*. In general, there were greater reductions in after hours utilisation in stand-alone services than embedded services in this MBS study, the opposite being true in the population survey study.

These results need to be interpreted in the light of the limitations and differences of both databases – the self-reported nature of the population survey but very importantly the absence of non-emergency after hours usage in the MBS database. While the two data collection methodologies focus on after hours GP care, they are capturing quite different service usage.

These Australian findings, presented for the first time, are generally consistent with the conclusion of the structured review on after hours care – namely that the growth in telephone triage and advice services usually, but not always, reduces immediate medical workload through the substitution of telephone consultations for in-person consultations [5].

In considering the policy significance of these findings, it is relevant to note that after hours service provision has moved on in Australia – as it has in other countries – since the conduct of this study with the introduction of second and third phase trials and the recent decision by COAG to fund a National Health Call Centre Network provided by nurses on a 24 hour basis [8] and the launch, for example, of Nurse-On-Call in Victoria.

These results need to be considered in conjunction with the results from other studies in this series. [4,7,9]. The first of these, the structured review concluded that the beneficial effects of telephone triage services in reducing immediate medical workload has to be balanced with the finding of reduced patient satisfaction when in-person consultations are replaced by telephone consultations. This may partly explain some inconsistent results for the stand-alone call centres that were established as a new type of service aimed at better addressing population needs. These inconsistent results include that

• the stand-alone services are clearly well used (results indicate that like the other triage services there is a non-significant trend to reduce unmet population need for after hours care)

• access to after hours GP services in the stand-alone services, however measured, did not improve; and

• results for utilisation effects of their services on GP after hours usage, unlike for embedded services, are not consistent for population and MBS datasets.

## Conclusion

The findings in general indicated that the telephone triage and advice services provided by the AHPMCTs usually, but not always reduced GP emergency after hours care. It is possible that other events and circumstances could be responsible for some of the reduction in after hours service use. These include other services opening in one AHPMCT area and new financial arrangements for AHPMCT care in another. The goals of stand-alone call centres to meet population needs may be being met only in part. Being demonstration programs, at least in the Australian context, these goals may be more fully met over time.

## Methodology

### Study design

The national evaluation constituted 'multiple trials' with common questions and hypotheses, rather than a 'multicentred' trial with common protocols [1]. This reflected the underlying goals of the AHPMCTs which were to meet local circumstances and needs, and to investigate different models and types of after hours PMC care most suitable given these local circumstances. The individual trials were therefore studied separately. While they are not directly compared, their relative success is considered.

A monitoring strategy was adopted, using a pre-post design to detect changes in relevant variables in the trial area across the study period. To examine whether these changes could reflect period effects occurring nationally, changes occurring in the trial area were compared with those occurring in the rest of Australia outside of AHPMCTs areas (the *National Comparator*).

### Data collection

#### MBS data

MBS data from the HIC was provided by the Department of Health and Aged Care (as it was then named) for after hours and in-hours GP services for all States and Territories. After hours was defined by the HIC as 8 pm-8 am weeknights, 1 pm–12 pm Saturdays and all day Sunday and public holidays.

1 *After hours items *(1, 2, 97, 98, 601, 602, 697 and 698). Their use depends on whether services were provided by Vocationally Registered (VR) or non-VR GPs, on whether they were provided in the GP's consultation room and whether they were provided in unsociable hours (11 pm–7 am) or not – see Table 2 for details. MBS after hours items cover only emergency (GP first call-out) not routine after hours care, as previously noted.

2 *In-hours items *(3, 4, 19, 20, 23, 24, 25, 33, 35, 36, 37, 38, 40, 43, 44 and 47). These items contain 96% of services in the General Practice Profile developed by the Australian Department of Health and Aged Care [10].

For States where the trials took place, the data was divided into two categories: trial areas and non-trial areas. For States where no trials took place, the whole state was deemed to be a non-trial area. Patients and GPs could be assigned to the trial and non-trial areas using residential postcodes for patients and practice location for GPs.

Monthly data for these MBS items was collected for the 3-year period November 1997 (when new after hours MBS items were introduced) to December 2000 (allowing time for full collection of data at the end of this period). Consultation rates are reported for GPs practising in trial areas and all their patients irrespective where they resided.

A 'cashing-out' arrangement was put in place for the *Deputising Service *– after hours GPs were paid on a salary basis and received no fee for a home visit. No claim by the patient or GP was made therefore for reimbursement (payment) from the HIC. These services therefore were not recorded and counted in the MBS database. They were counted separately under arrangements made with the trial operators.

#### ED and ambulance data

Data collection was attempted in all AHPMCT areas. However high quality data was only available for the *Regional Call Centre *and even here there were problems in the *Metropolitan area *in this trial as ED attendances that were recorded, included attendances at the GP after hours walk-in clinic operated by the *Regional Call Centre *adjacent to the ED for part of the study period – see Table 1. It was not possible therefore to conduct a useful analysis of the impact of the AHPMCTs on ED and ambulance service usage.

### Data analysis

Monthly total after hours GP utilisation rates per 10,000 population for both pre-trial and trial periods were compared in each AHPMCT area. Changes in each site were also compared with the *National comparator *(over the same monthly periods) to control for the effects of secular trend – see Table 9. Poisson regression analysis was used as the data consisted of counts of relatively rare events in a cohort of subject over a defined time period. The principal independent variables (additional to the constant) were: time (pre/during trial), intervention status (trial area/National comparator), and trial effect (time*intervention status). This generated a relative rate (and 95% confidence intervals) for the effect of the AHPMCT, expressed below:

**Table 9:** Impact of AHPMCTs on GP After hours utilisation rates*.

	**PRE TRIAL**			**TRIAL PERIOD**		
	**Period (Months)**	**Average**	**95% CI**	**Period (Months)**	**Average**	**95% CI**

**Statewide Call Centre (Metropolitan)**	16	19.3	14.8 – 23.8	20	15.9	12.9 – 18.8
National Comparator	16	30.1	23.6 – 36.6	20	28.3	23.2 – 33.4
Relative Rates	0.87 (95% CI 0.86 – 0.88)					
**Statewide Call Centre (Non-metropolitan)**	33	14.2	4.6 – 23.8	3	8.4	4.7 – 12.2
National Comparator	33	29.1	23.0 – 35.3	3	28.8	28.6 – 29.0
Relative Rates	0.60 (95% CI 0.54 – 0.68)					
**Regional Call Centre (Metropolitan)**	31	6.7	4.5 – 8.8	5	7.1	5.2 – 8.9
National Comparator	31	29.3	23.4 – 35.5	5	28.0	25.3 – 30.6
Relative Rates	1.11 (95% CI 1.06 – 1.17)					
**Regional Call Centre (Non-metropolitan)**	31	11.5	0.4 – 22.6	5	5.1	1.7 – 8.4
National Comparator	31	29.3	23.4 – 35.5	5	28.0	25.3 – 30.6
Relative Rates	0.46 (95% CI 0.35 – 0.61)					
**Deputising Service**	24	38.5	27.4 – 49.5	12	23.0	14.8 – 31.3
National Comparator	24	29.9	24.2 – 35.6	12	28.4	23.1 – 33.7
Relative Rates	0.62 (95% CI 0.61 – 0.64)					
**Local Triage service**	24	48.7	26.2 – 71.2	12	45.8	31.2 – 60.4
National Comparator	24	29.9	24.2 – 35.6	12	28.4	23.1 – 33.7
Relative Rates	1.01 (95% CI 0.94 – 1.08)					

* There are minor discrepancies in rates presented in Tables 2 and 3 arising from different time periods being used.

Relative rate (RR)=UTA.T1/UTA.T0UNC.T1/UNC.T0
 MathType@MTEF@5@5@+=feaafiart1ev1aaatCvAUfKttLearuWrP9MDH5MBPbIqV92AaeXatLxBI9gBaebbnrfifHhDYfgasaacH8akY=wiFfYdH8Gipec8Eeeu0xXdbba9frFj0=OqFfea0dXdd9vqai=hGuQ8kuc9pgc9s8qqaq=dirpe0xb9q8qiLsFr0=vr0=vr0dc8meaabaqaciaacaGaaeqabaqabeGadaaakeaacqqGsbGucqqGLbqzcqqGSbaBcqqGHbqycqqG0baDcqqGPbqAcqqG2bGDcqqGLbqzcqqGGaaicqqGYbGCcqqGHbqycqqG0baDcqqGLbqzcqqGGaaicqGGOaakcqqGsbGucqqGsbGucqGGPaqkcqGH9aqpdaWcaaqaaiabbwfavnaaBaaaleaacqqGubavcqqGbbqqcqGGUaGlcqqGubavdaWgaaadbaGaeGymaedabeaaaSqabaGccqGGVaWlcqqGvbqvdaWgaaWcbaGaeeivaqLaeeyqaeKaeiOla4Iaeeivaq1aaSbaaWqaaiabicdaWaqabaaaleqaaaGcbaGaeeyvau1aaSbaaSqaaiabb6eaojabboeadjabc6caUiabbsfaunaaBaaameaacqaIXaqmaeqaaaWcbeaakiabc+caViabbwfavnaaBaaaleaacqqGobGtcqqGdbWqcqGGUaGlcqqGubavdaWgaaadbaGaeGimaadabeaaaSqabaaaaaaa@6092@

Where U_TA.T1 _is the after hours utilisation rate per 10,000 population per month in the Trial Area (TA) during the trial

Where U_TA.T0 _is the after hours utilisation rate per 10,000 population per month in the Trial Area pre-trial

Where U_NC.T1 _is the after hours utilisation rate per 10,000 population per month in the National Comparator (NC) area during the trial

Where U_NC.T0 _is the after hours utilisation rate per 10,000 population per month in the National Comparator area pre-trial

SPSS for Windows version 12.0.1 was used for the analysis.

## Competing interests

The author(s) declare that they have no competing interests.

## Authors' contributions

DD made a substantial contribution to conception and design, acquisition of data, analysis and interpretation of data and was involved in drafting the manuscript. SED made a substantial contribution to acquisition of data, analysis and interpretation of data and was involved in revising the manuscript critically for important intellectual content. RW made a substantial contribution to acquisition of data, analysis and interpretation of data. MK made a substantial contribution to data analysis and was involved in revising the manuscript critically for important intellectual content. LG made a substantial contribution to data analysis made a substantial contribution to conception and design and was involved in revising the manuscript critically for important intellectual content.
